# Access to energy sources in the face of climate change: Challenges faced by women in rural communities

**DOI:** 10.4102/jamba.v10i1.375

**Published:** 2018-04-11

**Authors:** Mphemelang J. Ketlhoilwe, Kennedy M. Kanene

**Affiliations:** 1 Department of Languages and Social Sciences Education, University of Botswana, Botswana

## Abstract

Access to energy is a challenge to rural communities, especially among women who are the prime household energy users. This article is based on research carried out in the Tswapong villages in Botswana where energy sources particularly wood, are slowly getting depleted while electricity connection costs remain unaffordable for the poor. The article provides constructivist analysis of experiences in real-life situations among women. Data were generated through observations, documents analysis, interviews and focus group discussions. It has emerged from the research that majority of the respondents use firewood as energy source. Firewood and gas are mainly used for cooking while electricity is mainly used for lighting. The demand for firewood has led to firewood commercialisation, the depletion of preferred firewood tree species and increase in the impact of climate change. The article recommends economic diversification and subsidies to empower the majority of the rural poor to connect to the national electric grid and reduce on firewood dependence. These could be complemented by harnessing of solar energy and low-cost, energy-saving technologies. Subsidies to enable women access to energy services would contribute immensely to the decade of Sustainable Energy for All and to the attainment of the post 2015 sustainable development goal on energy.

## Introduction

While there are ‘debates about how to move towards less energy intensive lifestyles’ (Butler, Parhill & Pigeon 2014:2), there are some communities who are still faced with energy poverty and access issues. The overall focus of this article is on access to energy resources and services in the face of climate change. The article provides contextual background to illuminate the global and regional situation leading to local context in Botswana. It provides the experiences of communities concerning access to energy sources and services to indicate the economic challenges faced by some women in the rural areas. Omari (2010:25) notes ‘It is widely recognised that improving access to affordable energy services is a prerequisite to achieving economic growth and poverty reduction’. Affording women access to energy sources and services has the potential of achieving universal energy access and mitigating climate change.

## Contextual background

International Energy Agency (2012) reports that, globally, around 40% of the world’s population still rely on traditional biomass to meet household cooking needs. Besides, 70% of the rural population in the sub-Saharan Africa rely on traditional biomass. As such, most women and children spend hours collecting firewood for domestic use like cooking, heating and lighting. Some rural households use kerosene lamps for lighting that may contribute to pollution. Besides, an average of 540 mg per hour for wick lamps and 300 mg per hour for enclosed lamps is emitted (Practical Action 2010:3). Furthermore, pollutants from the cheapest kerosene wick stoves have the smallest particle size and are thus the most dangerous because they are taken more deeply into the lungs. Also, people stand the risk of getting affected by smoke when preparing meals and while making fire or sitting around fire to warm themselves (Practical Action, 2013). In some cases people use rudimentary stoves or agricultural residue to produce energy for domestic requirements. These are likely to cause respiratory-related illnesses. Meyrick (2014) noted that:

Exposure to smoke from traditional cook stoves and open fires causes 1.9 million premature deaths annually, with women and young children the most affected. Reliance on biomass for cooking and heating, also often forces women and children to spend long hours each week collecting fuel, with increased pressures on local natural resources. (p. 1)

Fraser (2012) reports that a quarter of global soot emissions can be traced back to cook stoves.

### Climate change and energy

The United Nations Secretary-General made important remarks at the Fourth World Future Energy Summit regarding our decisions on energy. He (Ki-Moon 2011) remarked:

The decisions we make today on energy will have far-reaching consequences. The prevailing fossil fuel-economy is contributing to climate change – and global energy needs are growing rapidly. In 20 years, energy consumption will rise by 40 per cent, mostly in developing countries, where 1.6 billion people still lack access to electricity, and where 3 billion people rely on traditional biomass fuels for cooking, heating, and other basic household needs. Our challenge is transformation. We need a global clean energy revolution – a revolution that makes energy available and affordable for all. This is essential for minimizing climate risks, for reducing poverty and improving global health, for empowering women and meeting the MDGs, for global economic growth, peace … (p. 6)

This clearly demonstrates the concern about access to sustainable energy and how women in particular are potential victims of climate change on energy resources. Sustainable development may not be possible without access to clean, affordable and sustainable energy for the poor. Therefore, policies and strategies on energy security should be geared towards improving access to energy services for all. Besides, the United Nations has designated 2014–2024 as the decade of Sustainable Energy for All. Sadly (UNDP 2008):

Still, one person in five on the planet lacks access to electricity and nearly 3 billion people are using wood, coal, charcoal, or animal waste to cook. Access to modern energy services … is a vast area of unmet need. The energy access challenge is particularly acute in the least developed countries. (p. 41)

Globally, about 1.2 billion people still lack access to electricity (World Bank 2013).

Collecting and using firewood contributes to deforestation, especially in climate change–affected areas where rains have failed or is inadequate. Energy issues may also contribute to climate change through emissions from burning solid fuels in open areas and traditional stoves because of incomplete combustion (Practical Action 2010).

### Local contextual issues

Access to commercial energy in Botswana’s rural areas (Aurela 2016):

is low with the majority of the population depending on non-commercial energy, mostly biomass. Access to electricity stood at 49% in 2008. Botswana has relied on imports to meet its growing demand for electricity. (p. 5)

However, solar energy potential is high, at 6.1 KWh/m^2^ per day. Other sources of energy such as wind, hydrothermal and geothermal are low. The main policies concerning energy sector include (Aurela 2016):

Vision 2016, National energy policy, Botswana Energy Master Plan (1996, reviewed 2003). Target is to reach 80% national power access and 60% rural access by 2016. Improved access, security and reliability of energy supply to all sectors of the economy, particularly the low income and marginalized. (p. 2)

Tswapong villages are located in the eastern part of Botswana. The villages lie to the east of the main railway line from north to south, in Serowe-Palapye sub-district and administrative region. There are over 26 villages scattered along the foothills with a total population of over 68 000 inhabitants mainly subsisting on arable agriculture and livestock farming. The area depends on unreliable rainfall that averages between 250 mm and 450 mm per annum. Although almost all the villages have access to the main national electricity grid, not all families afford connections to their homestead because of their economic status. They depend on firewood collected around the settlements and distant areas. The majority of these families are women. This research focused on women in particular because they are more affected by energy poverty.

Issues that affect women and energy remain topical in the face of poverty and climate change impact. Rural women in the Tswapong villages use firewood for their energy requirements in the home largely because they are unemployed and poor and have heavy burdens as family heads. Through government departments, non-governmental organisations and the media, women are exposed to different ways of making a living including empowerment to be independent through use of social and entrepreneurial skills. To enhance this, these women require knowledge on the natural resources they could obtain for free from the wild as well as energy resources to process natural resources into commodities for sale and consumption (Ketlhoilwe & Jeremiah 2013). According to Omari (2010), in Botswana:

females are most involved in fuel wood collection and they spend over 3 h a day on average collecting fuel wood … time spent collecting firewood is expected to increase as resources become scarcer; this is a possible impact of climate change. (p. 25)

When a household is unable to afford purchasing firewood, the burden usually falls on women and/or children to collect firewood. In most villages they collect wood from distant areas without transportation. That means firewood collection has become labour intensive and time consuming with low results as women and children can only carry so much weight on the head or shoulders for a long distance. Studies have shown that men are only involved in firewood collection when there is transport like a vehicle or donkey cart (Omari 2010). Moreover, men often get involved in firewood collection as an income-generating activity.

Fuel wood accounts for 43% of the final energy consumption in the rural areas in Botswana (Wright 2007). Access to electricity is low, 31% in rural areas. As rural population increases and land-use activities intensify, more woodland gets degraded and as a result, sources of wood and collection becomes a challenge. Eighty-two per cent of the Serowe-Palapye inhabitants use firewood for heating, while only 16.1% use electricity. ‘The implications for excessive harvesting and use of firewood for heating are … elevated risks of deforestation, land degradation and desertification, and contamination of the environment’ (Statistics Botswana & UNFPA 2015:10).

Wood sources, particularly tree species that are traditionally used for firewood, have become more scarce in the village neighbourhood because of overexploitation as firewood gets commercialised by those with the means to transport wood from distant areas. Because of firewood commercialisation, some wood collectors end up cutting live trees, leading to land degradation and associated effects such as deforestation and desertification contributing to adverse impact of climate change.

The Botswana Government ‘overall policy goal for the energy sector is to provide affordable, environmentally friendly and sustainable energy services in order to promote social and economic development’ (Government of the Republic of Botswana 2009:5). The national rural electrification project has seen electricity grid distribution to most parts of the country. In addition, the Government has introduced a subsidised rate of P5000 per household for electricity connection as a policy intervention. However, the majority of the rural poor especially women are still unable to afford the subsidised rate for home electricity connection.

The challenges affect those dependent on firewood for almost all their energy requirements as they get disproportionately denied access to the resources. In Botswana ‘women are the major users and collectors of household energy fuels which predispose them to environmental problems brought on by exposure to traditional fuels, including indoor pollution’ (Omari 2010:33). Majority of the women from the research sites use wood for preparing daily meals, *morula* and *lerotse* (cultivated mellon) jam, for boiling herbs, cooking and water heating.

### Impacts related to access to energy

Inadequate or no access to energy services and systems may lead to household energy poverty especially among women who have no formal employment or reliable sources of income for household requirement or any domestic and productive tasks that require energy use. Energy poverty in this article refers to having inadequate access to energy services for basic household needs and for income generation. Inadequate access to energy has an impact on sustainable development among the affected communities. People spend more time collecting firewood, sitting around fire warming themselves and hence increasing demand for firewood. Time spent on these activities increases the levels of poverty because it could have been devoted to socially or economically productive activities such as education or agricultural production. Collecting firewood has been found to be affecting poor women’s health. Each year, 4.3 million pre-mature deaths, mainly of women and children, are linked to toxic fumes from fuels such as wood, animal waste and charcoal used for cooking and heating (SE4All 2015). In addition, small enterprises can be severely affected by rising energy costs, fuel shortages and deforestation.

## Conceptual framework

Conceptually, the article is guided by a constructivist approach framed within the belief that reality is socially constructed. Constructivist approach is based on the premise that knowledge is constructed through the interaction of people and the objects of inquiry (Lotz-Sisitka, Fien & Ketlhoilwe 2013). In this research the researchers deployed this tradition through interaction with respondents during observations, focus group discussions and interviews in the research sites. This is a qualitative study involving ideas about powerlessness among women in respect to energy access and acting both individually and collectively to change the conditions of living (Lee et al. 2013; Lather 1991). Data generated from this research assisted in identifying and analysing individual and group constructions and interpretations of reality as experienced by women in the Tswapong villages.

## Aim and objectives of the research

The research aim was to explore access to energy services by women in the rural Tswapong area in the eastern part of Botswana. The objectives that guided the research to accomplish the above aim were the following:

- to investigate the sources of energy
- to explore access to energy services by rural women in the research sites
- to examine the risks and threats related to energy poverty among the users
- to investigate women’s resilience strategies in mitigating the risks and threats.

The answers to the above aim and objectives contributed to understanding, deconstructing and to voicing an emancipatory approach to gendered energy poverty. It has been noted that (Lee et al. 2013):

studying women’s experiences, we open up new empirical and theoretical resources and provide a new purpose for social science (women rather than men), and a new subject matter of inquiry. (p. 380)

## Research methods

The central question that this qualitative research sought to answer was on access to energy services in the face of climate change. To answer this question, a detailed understanding of women’s interaction with their immediate environment placing it within the specific context of rural women in Botswana was carried out. Data were generated through semi-structured interviews, participant observation and focus group discussions as well as insights from published and unpublished literature, and other available sources of information. Data were subjected to qualitative processing and analysis for emerging categories as presented in the main findings.

### Interviews

Interviews were conducted to gain an understanding of issues from the perspectives of the interviewees. Semi-structured interviews were used mainly to understand the general perspective of women about the resources therein and challenges to access to energy sources. Semi-structured interviews involved discussion on many issues from specific access to energy sources and services, livelihood activities such as farming or use of natural resources. These were also triangulated by using information generated through focus discussions. Participants of semi-structured interviews were of different age groups including those over 65 years. Rich data were acquired through this method as respondents did not perceive the interviews as questioning sessions but a curious interest in their lives by outsiders. However, when in the field, in many instances more probing had to be done in order to keep engaging the interviewees on access to energy experiences. The semi-structured interviews were found to be useful as they allowed for focused, conversational, two-way communication, and most importantly, it allowed for a fairly open framework which was used both to generate and receive information. Interviews were useful as they assisted in collecting household-level economic data which may have been complex without using this method. These interviews enabled the researcher to deploy the constructivist approach where participants freely articulated their experiences reflecting the reality on the ground.

### Focus group discussions

Group discussions were used mainly to acquire information on access to energy sources and services. This required detailed preparation of a checklist of open-ended questions on specific issues surrounding the energy activities being studied. For instance, detailed focus group discussions were held with a group of 10 small-scale commercial egg production women who are members of the Chadibe Poultry Project. Most of the respondents therefore were women who stay in the village, often unemployed and caring for the young and elderly as well as for the dry land field crops. The discussions assisted in obtaining the general understanding of important issues concerning energy in the rural areas, particularly on how it affects their poultry project production and household energy requirements. It helped in gaining in-depth insights on how women are coping with the impact of climate change on energy sources. A social constructivist approach guided the open-ended questioning to understand the complexity of the research participants’ views on energy sources within their area and households (Creswell 2009). The discussions assisted in knowledge co-constructions with the research respondents.

### Document content analysis and literature review

This was carried out throughout the research process. That is, before, during and after the fieldwork. Policy and other official documentation gave insights into energy, gender and natural resource management and use issues. This process helped shape the questions to ask and generate data that could otherwise not be gained in the field because most respondents did not have this information. The aim of analysing the documents was to generate data that would enhance the researchers’ understanding of women, climate change and energy systems experience in the rural areas. The literature in a way strengthened qualitative information on experiences of rural women on access to energy sources and services.

### Participant observation

This method was carried out in the study areas in order to make informed conclusions, gain some insights into the lives of the respondents and their interaction with each other, energy services and resources around them through observations. Visits were made to participants’ homes, firewood collection sites and projects. This proved very useful in understanding the local context. A scheduled visit was done to the Sefhare Village where an oven for baking bread was handed over to a group of women. This helped the researcher to appreciate what it takes to be in an area where firewood is becoming scarce and other sources of energy are not easily accessible. Interaction with women from different villages helped appreciate the challenges faced in trying to collectively or individually to alleviate energy poverty among women. Another observation activity was in the Lerala Village where women were processing and packaging natural herbs and marula oil. The method helped the researcher to explore social and livelihood complexities and to appreciate the need for energy within the factory. Women use the cold press method to extract oil from marula kernels. The process requires manual labour and it is a challenge even to the able-bodied women. It makes the oil production slow and low. It is time–consuming as it was observed that it takes about a day’s work to produce a litre of oil. However, the cold extraction method is still preferred as it produces quality natural marula oil preferred by customers. These observations assisted in the interpretive analysis of women’s access to energy services and experiences.

Observations improved the researchers’ understanding, triangulation and validation of data regarding the use of firewood that were generated through mixed methods. In analysing data, it was important to look at deconstructionist’s impulse that provokes considerations for the gendered positions made available by women’s voices of their experiences regarding access to energy services in the rural areas. The analysis was based on women’s perspectives; that is, their construction of the reality on access to energy sources as they experienced it.

## The findings

Data for this researched case study article were generated from 10 villages in the Tswapong region in the eastern part of Botswana located within the Serowe-Palapye sub-district (Statistics Botswana & UNFPA 2015). There were 493 respondents in all out of a total of over 35 000 inhabitants of the 10 villages, of which 19 249 are female residents and 7708 are between the ages of 15 and 64 years. The interviewed women include individuals (120) and focus group members (373) in the research sites. Some of the focus group members were also interviewed individually to validate data. The respondents were of varied age groups, all of them were not formally employed. The majority (85%) practice subsistence farming, supplementing it by gathering wild products to support their families.

### Energy sources and types

Poor rural households exploit natural resources, wood in particular, leading to reduction or loss in natural capital that eventually affects them. The research participants identified firewood (88%), electricity (8%) and gas (4%) as sources of energy for cooking as shown in Table 1.

**TABLE 1:** Energy sources and types.

Energy source	Frequency	Percent
Firewood	434	88
Electricity	39	8
Gas	20	4

Noteworthy from Table 1 is the fact that each research participant was at liberty to identify multiple responses regarding the source and type of energy for cooking.

### Modes of firewood acquisition

The study sought to investigate the modes of firewood acquisition among the respondents who use firewood as a source of energy. The research participants indicated that they collect wood from the bush (59%), some (29%) buy wood, while others collect and buy wood (12%) as indicated in Table 2.

**TABLE 2:** Mode of firewood acquisition.

Mode	Frequency	Percent
Collecting from the bush	256	59
Buying	126	29
Collecting from the bush and buying	52	12

**Total**	**434**	**100**

Interestingly, even those who are using gas and electricity sometimes use firewood especially when out of gas or during electric power cut. Wood is obtained from distant areas mostly by carrying it on the heads. It was also confirmed through observation and discussion that wood sources are becoming scarcer and require transport and more time for the collectors. Some people use donkey carts or vehicles to travel long distances in search of firewood. From the source, wood requires a lot of labour to cut it into the required pieces and also local knowledge and skills to identify tree species that are good for making fire; that is, those that produce more heat.

Research respondents who use electricity indicated that they are connected and buy tokens for the pre-paid electricity from the local vendors. Women’s main challenge is lack of money to either consistently pay for electricity or connecting it to their houses. It was observed that some respondents (87%) use firewood for heating while 89% uses firewood for daily preparation of meals compared to 25.9% of the Serowe-Palapye sub-district (Statistics Botswana & UNFPA 2015). Some women use paraffin (28%), candle (4%) and electricity (30%) for lighting purposes in their houses.

In preparing natural products such as marula jam, women use firewood (4%) collected from the bush and gas stoves (6%). Gas stoves are usually used once firewood is depleted as it is more expensive than wood. Other activities that need energy are making local brew for sale (24%); making a traditional vegetable dish (morogo) (12%) and making bread and fresh potato fries (8%). A summary of data on the responses of the women regarding uses of wood energy is shown in Table 3.

**TABLE 3:** Uses of wood energy by rural women.

Use	Frequency	Percent
Heating	429	87
Preparation of meals	439	89
Making local brew	217	44
Preparing jam	20	4

Women felt that collection of firewood is affecting the environment leading to depletion of trees such as mophane tree, causing soil erosion (6%) and land degradation (12%). The environmental impact is with respect to the economic, health and environmental aspects.

#### Economic issues

If energy is obtained for free in traditional setting, it would help women spare money. Some of the women interviewed are now concerned as they are using money to buy firewood obtained far from their villages and homes. They are constrained in preparation of food and natural products that need energy. There is the cutting of live trees as a result of deadwood depletion and this may cause land degradation leading to poor yields and poor pasture and hence poverty in the affected areas. The cutting of live mophane trees lead to low yield of mophane caterpillars which is a source of livelihood for the local people as a local relish and is often sold to commercial farmers and retailers.

To build resilience and cope with energy poverty, some of the respondents have managed to secure tenders to supply local primary schools with fresh homemade bread. For instance in Ratholo village, two women were supplying a local primary school with fresh bread while in Sefhare Village, a group of women have managed to build an oven for baking bread for sale to schools and the public. However, both groups use firewood for baking bread. These activities are likely to diversify the economy of women in the Tswapong region contributing to community resilience to both adverse impact of poverty and climate change. The challenge women face in diversifying the economy includes that of access to energy sources such as electric power and gas.

#### Health-wise and social issues

Some women felt the use of firewood in products preparation sometimes caused ‘burns to the skin, high blood pressure and smoke affects our eyes’. They suggested the use of electricity or gas stoves as alternatives. Some women complained that after carrying a bundle of firewood on their head they experienced body pains.

Traditionally, firewood was collected by women who were socially bounded by often interaction and living within the proximity to each other. This arrangement facilitated social learning among women. Women learnt firewood species and taboo associated with collecting the wrong wood species for use. They also learnt from each other that ‘dry wood was suitable for use especially for cooking rather than wet wood that may produce more smoke and smell’. Women were concerned that ‘proper socialization is now lacking as people now go on their own to collect firewood’. One woman said ‘in the past we used to counsel each other, share ideas, and solve emotional issues during wood collection’. The practices of firewood collection in groups used to encourage friendship. This practice is no longer common as firewood is obtained far and some people have better means of transport to go far distances to collect firewood for use and sale. The social learning process among women in rural areas has now declined and ‘some young women collect wood that they do not even know the name of the tree from which it originates’. This results in women using wood from trees that are a taboo to use for making fire.

#### Environmental issues

Climate change has affected annual supply of firewood as a source of energy for households as well as small-scale entrepreneurial activities as all of them are dependent on wood use for cooking, water heating and natural products processing. The research respondents had identified some ways in which climate change has affected wood supply. One respondent said ‘we are experiencing extreme heat and rain has become less frequent, this may lead to wood depletion in the long run’. While another respondent said ‘there is limited regeneration growth among important firewood tree species as a result of varied rainfall pattern’. One woman said ‘we experience heat stroke and some trees may also be getting affected by heat’. Another woman said ‘rainfall pattern is not normal anymore and it is sometimes too heavy and destructive to the vegetation including trees used to obtain firewood’, and one said ‘there are no enough trees for firewood, people cut life trees excessively and without rainfall there is less regeneration of vegetation including trees’.

## Discussion

The majority of women in the rural areas use firewood for cooking and heating (CSO 2001; Omari 2010). Firewood is also used for small-scale jam preparations and other commercial products that need heat. Firewood is becoming inadequate because of high demand within villages and from the neighbouring towns. It is, however, the main source of energy for the rural majority. In the past, firewood collection was done on almost a daily basis by women forming some social groups. This practice is no longer common as people are becoming more individualistic as firewood is obtained from distant areas and has become commercialised, thus affecting socio-economic practices. These affect the rural energy service negatively as majority of the people, especially women, are not employed and hence have no income to purchase wood. Firewood collection leads to environmental degradation. Some respondents noticed this and said ‘it is causing soil erosion as areas get bare and degraded’. Those who can afford alternative sources are supplementing the use of firewood for cooking. As for lighting, they use electricity and paraffin which they report to be expensive for them to buy. Despite the fact that electricity connection to individual household is subsidised, majority of the rural poor are still not able to afford their required contribution. As a result, they keep looking for free or cheaper energy sources such as firewood. However, households that have connected electricity continue using firewood as it is cheaper and helps them reduce on electricity bills. The research respondents claimed that ‘paraffin has become expensive and is often in short supply despite the introduction of the solar lamps and battery charged lights’, which they cannot afford to buy and maintain. This leaves poor people with limited alternatives for preparing meals, heating and providing light.

Energy poverty compromises sustainable development efforts, especially among those rural dwellers who are unemployed and depend on arable farming and government intervention policies. Women’s access to energy may increase economic efficiency and productivity gains with less time and physical exertion spent on basic subsistence activities such as firewood collection. Access to energy may create entrepreneurial opportunities and new markets for private investors, particularly micro, small and medium-sized enterprises owned by women (Schalatek & Burns 2013).

Despite the Government’s effort to connect villages and households to the grid, some women ‘continue to collect and use firewood to cook and boil water for their families’ (Wright 2007:41). This inability of rural women to afford electricity connections can be resolved by financial mechanism to allow poorer households to pay connection fees; this would not only provide benefits to women-headed households but would also improve profits by increasing the overall connection rate (Schalatek & Burns 2013). Connections to clean energy sources would create ‘multiple-win’ solutions with economic, development and gender equality co-benefits while significantly reducing greenhouse gas emissions. Above all, making clean energy connections to rural households would reduce on energy poverty. Improving access to energy is becoming more relevant and in demand as a means of improving the lives and livelihoods of the people. It would also transform the local economies and influence the production and consumption pattern in the rural areas for long-term sustainable development goal.

Majority of the research respondents are dependent on climate-sensitive resources (Kapoor, Rai & Chowdhury 2011) such as those in the farming sectors and natural products. During droughts they depend more on drought-resistant natural products and crops. However, all these become inadequate during droughts. It has emerged that, as a result of climate change and variability in the area, rainfall is becoming more unreliable and temperatures are becoming more intense, leading to wilting of the crops and wild plants. This indicates that the common capital is affected. As a result, poverty increases, adding to energy poverty. To build resilience to both poverty and climate change impact, a member organisation called Kgetsi-ya-Tsie is empowering its membership with some entrepreneurial skills and encourages them to take advantage of national policies and strategies meant to reduce poverty (Ketlhoilwe & Jeremiah 2013). It emerged from the study that most of the women who are members of community-based organisation depend on firewood for cooking and heating in their homes. Some of them would not be able to prepare something to eat without firewood. Overcollection of firewood occurs especially around villages leading to deforestation that contributes to global warming, leading to climate change. This situation is made worse by persistent and high demand for commonly used firewood tree species. As a result of inadequate supply of firewood, the majority of women are left with only two options to get firewood. They either have to travel long distances or to buy from those with donkey carts. While travelling long distances to collect firewood remain a challenge, their purchasing power is even lower during drought years when yield from the field crops is low or their domestic animals could not be sustained as a source of income to purchase firewood. These situations contribute to energy poverty, especially among women-headed household.

Any effort to introduce electricity use among the women household should be accompanied by appropriate technologies and education to promote energy efficiency. Awareness on lowering household energy consumption and wise use should be one of the focal messages. Access to appropriate, renewable and sustainable clean energy for the poor could also cater for reduction on emissions.

## Conclusion and recommendations

Access to affordable forms of sustainable energy is crucial to the needs of those at the base of the economic pyramid, especially women in rural communities (Ulsrud et al. 2015), for the achievement of Sustainable Development Goal 7. Majority of women in the rural areas in Botswana depend on traditional knowledge on appropriate wood species for fire making. Traditional knowledge that was socially constructed and acquired is an asset to sustainable development endeavours that need to be recorded and legally protected for future generations. Rural communities are dependent on firewood for cooking and heating. It is clear from the findings that sources of firewood tree species are threatened by population expansion and high demand from buyers, and wood is becoming commercialised. It is recommended that woodlots be introduced in affected areas for future firewood supply catering for families off the electricity grid. Low-emission energy investments that are gender-responsive could contribute to increasing women’s access to modern and clean forms of energy for lighting, cooking, heating and other productive uses. They could increase economic efficiency and productivity gains with less time and physical exertion spent on basic subsistence activities such as wood fuel collection, creating entrepreneurial opportunities and new markets for micro, small and medium-sized enterprises owned by women (Schalatek & Burns 2013). The use of more energy-efficient stoves by women and the general public could help reduce the depletion of wood resources. However, the cooking stoves should not only be energy efficient but also produce less smoke to minimise on local air pollution.

Climate change is not only affecting energy resources for energy production, it is also affecting food production and annual supply of veld products that women depend on. It is recommended that women be engaged more on entrepreneurial skills and forestry activities to build resilience to climate change. Some women in the research sites are engaged in poultry production, bread making and tuck shops. All these economic activities need efficient access to energy services and sources to be sustained and to reduce dependence on natural resources to promote contribution to energy poverty reduction and achievement of sustainable development goal 7 on affordable and clean energy.

The use of renewable energy is considered as an indispensable component of sustainable development (Uddin & Taplin 2008). Therefore, energy systems, appropriate strategies and institutional settings are needed to be in place for the poor and vulnerable communities. Access to energy sources and services by women would be one of the economic empowerment strategies that would not only reduce poverty but also accelerate sustainable development. Social protection systems need to be established to enhance the capacity of the poor and the vulnerable people to enable them to better manage risks and shocks (OECD 2012). This article argues that further significant effort could be made towards energy sustainability in Botswana. As Botswana is well positioned in sub-Saharan Africa within the tropics and receives sunlight most of the day times, a solar energy strategy could facilitate access to affordable and clean energy for the benefit of all including women in the rural areas. The mean annual solar insolation is reported to be ‘21 MJ per m^2^ per day, one of the highest radiation levels in the world’ (Government of the Republic of Botswana 2009:5). It has also emerged that given support, rural women are capable of empowering themselves and becoming resilient to poverty-related situations. Women use social learning approaches to vary the means of their ways of leaving. It is recommended that women in Tswapong area be assisted to use alternative sources of energy to reduce and ultimately avoid use of wood for making fire; use profit generated from women’s entrepreneurial projects to connect to national power grid to avoid the challenges regarding energy access. These would reduce adverse effects of climate change impact and hence reduce energy poverty.

The Botswana Government subsidy on electricity connection is a commendable intervention. However, more could be done to increase the subsidy making it a social investment as access and use of clean energy would contribute to public health savings by reducing the risks of exposure to biomass smoke and pollution. The introduction of affordable low energy-consumptive and emission-reductive technology (Sanaeepur et al. 2014) would assist poor families to adapt to the adverse impact of climate change. Finally, all efforts to promote access to clean energy in the face of climate change should be accompanied by education on the value of access to affordable forms of sustainable energy. Ensuring energy security and through sustainable energy systems policies would address social inclusion and gender equity concerns.
